# Efficacy of Mindfulness-Based Stress Reduction on Mood States of Veterans With Post-Traumatic Stress Disorder

**DOI:** 10.5812/atr.8226

**Published:** 2013-02-01

**Authors:** Abdollah Omidi, Abolfazl Mohammadi, Fatemeh Zargar, Hossein Akbari

**Affiliations:** 1 Trauma Research Center, Kashan University of Medical Sciences, Kashan, IR Iran; 2 Department of Clinical Psychology, Kashan University of Medical Sciences, Kashan, IR Iran; 3 Department of Public Health, Kashan University of Medical Sciences, Kashan, IR Iran

**Keywords:** Mindfulness-based Stress Reduction, Post-traumatic Stress Disorder, Veterans

## Abstract

**Background:**

Mood and negative emotional states and their regulation in patients with post-traumatic stress disorder have family, social and employment problems. Practices that could be helpful in this area are highly important.

**Objectives:**

The current study aimed to investigate the influence of mindfulness-based stress reduction (MBSR) in improving mood state of combat veterans.

**Patients and Methods:**

In this randomized clinical trial study, participants were selected from the patients referring to the counseling center of the veterans. The participants had post-traumatic stress disorder according to diagnostic and statistical manual of mental disorders, fourth edition, text review (DSM-IV-TR). Sixty- two patients were randomly assigned into 2 groups: (31 for MBSR and 31 for the control group).

**Results:**

Analysis showed that there were no significant differences between the groups at baseline (P < 0.05). Comparison of the results between the two groups before the two-step test showed that anger and vitality scales between the two groups have no significant differences, but on the other scales (depression, dizziness, fatigue and tension), differences between pre and post-test groups were significant in the two groups.

**Conclusions:**

It was concluded that mindfulness-based stress reduction is a useful method to regulate the mood state in veterans with post-traumatic stress disorder (PTSD) who have difficulties in mood and emotions in Kashan.

## 1. Background 

In recent years emotional regulation strategies have been considered in psychological problems. Post-traumatic stress Disorder (PTSD) is one of the most common disorders among veterans related to problems in emotional regulation. Common symptoms of PTSD include flashback episodes, emotional numbing, having a lack of interest in normal activities, excessive emotional arousal such as difficulty in concentrating, feeling irritable or having outbursts of anger and having trouble falling or staying asleep (1). Treatment of excessive emotional arousal can be effective in patients suffering from mood and emotional instability syndromes such as PTSD (2-5). Furthermore, mood states of veterans have reciprocal interaction with other symptoms of PTSD, improvement of their emotional state may affect recurrence and relapse of PTSD (5). Interventions based on mind and body such as mindfulness-base cognitive therapy (MBCT) (6) and mindfulness-based stress reduction (MBSR) (7) have positive impact on the problems caused by emotional arousal such as sleep disorders, restlessness and stress (8-10). Many studies have indicated that interventions based on mindfulness reduce stress and anxiety (11), chronic pain (12) and eating behavior (13). These interventions are examples of awareness training programs (ATP) that focus on the role of "mind training" in regulating mental and physical states (14). Studies have shown that these interventions can relieve the severity of a wide range of health problems help the mind to relax, identify the mind- body dysfunction and be aware of their connections. Using awareness and mindfulness techniques, people can develop coping with their problems. In addition, these techniques are easily learned and can be considered as available clinical interventions. Mindfulness is defined as a state of awareness and attention that occur in the present. Mindfulness helps clients to free themselves from the automatic thoughts, habits and unhealthy behavior patterns. Therefore, mindfulness plays an important role in regulating behavior (15, 16). MBSR is an eight-week educational course based on mindfulness techniques (6). This intervention makes standpoint of individuals separate from their cognitions and decant them. In these courses, people learn to observe their thoughts and feelings without judging them and see them simply as mental events. They learn that reality is different from their thoughts. MBSR is a class-based program to reduce stress problems.

## 2. Objectives

The current quasi-experimental research aimed to investigate the efficacy of MBSR on mood, emotional and behavioral functions of veterans with PTSD.

## 3. Patients and Methods

A randomized clinical trial design was used in the current study. Participants were selected by a psychiatrist from the patients referring to the counseling center of the veterans which was an active visiting center. The participants had post-traumatic stress disorder according to DSM-IV-TR diagnostic criteria and had treatment processes. Sixty- two patients were randomly divided into 2 groups: (31 cases and 31 controls). Then, written agreement of the patients for active participation was made. All patients were male, aged between 39 - 59 years.

### 3.1. Intervention 

Experimental group (MBSR): available scientific sources were employed to design the treatment protocol. The protocol consisted of two-hour group sessions in which cognitive exercises and meditation techniques were taught. Homework included meditation and other techniques on a daily basis during the 8 weeks (group program of mindfulness training developed by Kabat-Zinn) (7). Control group (TAU): this group of patients continued their routine treatment (antidepressants and anti anxiety by psychiatrist) without any more intervention.

### 3.2. Measures

1- Structured clinical interview for DSM-IV (SCID-I): the structured clinical interview for DSM-IV axis I disorders (SCID-I) (17) is a diagnostic exam used to determine DSM-IV axis I disorders (major mental disorders). The instrument is designed to be administered by a clinician or trained mental health professional. The SCID is broken down into separate modules corresponding to categories of diagnoses. Most sections begin with an entry question that would allow the interviewer to "skip" the associated questions if not met. For all diagnoses, symptoms are coded as present, sub threshold, or absent. 

2- Inventory of mood status (BRUMS): this questionnaire was presented by Terry in 1999 (18). It is a 24 item questionnaire which includes six subscales: anger, confusion, depression, fatigue, stress and vitality. A Likert scale was used for five groups as follows: Zero = no, 1 = slight, 2 = moderate, 3 = high, 4 = very much. Subsets of these six factors are: Anger factors which include disgruntle, Let anger, confusion, which includes turbulence and uncertainty. Fatigue and burnout factor - confusion - sleepiness and fatigue and stress factors which include panic - being anxious, worried and nervous, and finally a factor of life alive - energetic - that is active and alert.

### 3.3. Statistical Analyses

 First demographic differences between the MBSR course participants and those of TAU group were evaluated. Frequencies, mean values and standard deviations of all outcome measures were calculated. According to the central limit theorem, normalization of responses was not required and groups were independent, therefore, it seems preferable to use ANCOVA to evaluate differences in PTSD symptom severity between the two groups. To examine whether PTSD symptoms were associated with likelihood of volunteering for MBSR, a one-way ANOVA was conducted, with course participation as the independent variable and PTSD symptom severity as the dependent variable.

## 4. Results

Demographic characteristics are shown in Table 1. Analysis of variances indicated that there were no significant differences between groups at baseline (P < 0.05). There was no significant difference in PTSD symptom severity between those who did and did not volunteer for MBSR, (P < 0.05). This analysis was also conducted with the full baseline sample to ensure that the result was not the product of differential attrition. Again, there was no significant difference in PTSD symptom severity between those who did and did not volunteer for MBSR, ( P < 0.05). Table 2 shows the detailed results of mood states at pre and post tests in MBSR and TAU groups: comparison of results between the two groups before the two-step test shows that anger and vitality scales between the two groups had no significant difference, but in the other scales, differences in the two groups were significant between pre and post test.

**Table 1.** Demographic Variables in Experimental and Control Groups

Range	MBSR^a^ Group, No. (%)	TAU^a^ Group, No. (%)	P value
**Age, y**			0.956
35 - 39	8 (25.8)	7 (22.6)	
40 - 44	21 (67.7)	22 (71)	
45 - 49	2 (6.5)	2 (6.5)	
**Education**			0.5
Diploma	16 (51.6)	15 (48.4)	
Undergraduate	15 (48.4)	16 (51.6)	
**Occupation**			0.61
Occupied	22 (71)	22 (71)	
Retrieved	9 (29)	9 (29)	
**Marital status**			0.754
Married	30 (96.8)	30 (96.8)	
Single	1 (3.2)	1 (3.2)	
PTSD^a^ symptoms	16 (43)	17 (44.5)	<0.05

^a^Abbreviations: MBSR, mindfulness-based stress reduction; PTSD, post-traumatic stress disorder; TAU, control group

**Table 2.** Analysis of Co-Variance on Mood StatesBetween the Two Groups at Pre and Post Test

Variables	MBSR^a^, Mean ± SD		TAU^a^, Mean ± SD		P value
	Pre	Post	Pre	Post	
**Anger**	94.7 ± 17.8	82.2 ± 25.5	92.1 ± 15.1	87.6 ± 15.7	0.191
**Dizziness**	71.9 ± 13.2	60.9 ± 14.5	67.1 ± 31.1	71.8 ± 14.6	0.005
**Depression**	89.3 ± 20.1	72 ± 21.8	91.2 ± 23.2	92.2 ± 19	0.005
**Fatigue**	67.1 ± 9.7	60.2 ± 12.4	67.2 ± 20.1	71.2 ± 10.2	0.001
**Tension**	62 ± 9.4	54.5 ± 11.5	61.7 ± 15.8	63.8 ± 12	0.001
**Vitality**	45.5 ± 6.3	45 ± 11.6	41.9 ± 15.5	43.5 ± 7.1	0.638

^a^Abbreviations: MBSR, mindfulness-based stress reduction; TAU, control group

## 5. Discussion

The present study attempted to investigate the efficacy of MBSR on mood state of veterans based on a clinical trial experiment. The results of this study indicated that MBSR course has been able to reduce the rates of depression, dizziness, fatigue and tension in participants significantly.

Other studies on MBSR showed the effectiveness of this treatment on reducing the negative automatic thoughts, dysfunctional attitudes, depression and anxiety (19). The results of the current study were consistent with findings of Nakamura et al that showed MBSR was effective in some symptoms of veterans such as dizziness, depression, fatigue and tension (20). Also, MBSR could improve general mood of veterans because MBSR exercises increase awareness of the moment, affect cognitive system and information processing by techniques such as focusing attention on the breath and being in here and now (7). Mindfulness may be an important treatment of stress, increasing coping with stress and resiliency in veterans (8, 21). However, mental health professionals have recognized that mindfulness can have many benefits for people suffering from difficulties such as anxiety and depression (20, 22-24). No significant interactions were found between PTSD symptoms severity and treatment outcome. It means that MBSR is an effective treatment for a wide range of people dealing with painful affects and thoughts, including those suffering from trauma-related events. The findings should be interpreted cautiously, according to the small sample size, but they seem to be a promising intervention for future study. Therefore, according to the efficacy of MBSR and usefulness of this method in improving the quality of mood and emotions and subsequently improving the quality of life and mental health, widespread application of this method is recommended. The clinicians who are willing to save time and energy are recommended to use MBSR practices. The potential payoffs are substantial. MBSR incorporates elements of traditional, psychological, and relaxation techniques that elicit the relaxation response. This response reduces autonomic nervous system reactivity (25). Adding flexibility to one’s clinical repertoire of relaxation practices, MBSR provides an alternative means of achieving relaxation state. Gradually, MBSR practices help to alter essential physiological factors (breathing synchrony, heart rate, etc.) that are easily disrupted by stress and create a satisfying sense of physical integration. The deliberate slow pacing of mindfulness, as practiced in the MBSR program, provides a wonderful setting for mindfulness practice, offering a wealth of kinesthetic, proprioceptive, and interoceptive cues for focused attention. For many people (clients and clinicians alike), mindfulness offers an accessible means of cultivating psychologically and physically graceful patterns that not only feel good, but can help bolster feelings of well-being particularly among medical patients who often discover that they possess physical capabilities not previously utilized. And as a means of providing a unified body / mind experience, MBSR has few equals. Even brief periods of MBSR can help to create an atmosphere of relaxed congeniality that enhances the quality of therapeutic interactions.
